# Effects of context and discrepancy when reading multiple documents

**DOI:** 10.1007/s11145-022-10321-2

**Published:** 2022-06-28

**Authors:** Cornelia Schoor, Jean-François Rouet, M. Anne Britt

**Affiliations:** 1 University of Bamberg, Bamberg, Germany; 2 Leibniz Institute for Educational Trajectories, Wilhelmsplatz 3, 96047 Bamberg, Germany; 3 Centre National de la Recherche Scientifique and Université de Poitiers, Poitiers, France; 4 Northern Illinois University, DeKalb, USA

**Keywords:** Reading comprehension, Context, Consistency of information, Reading behavior, Multiple documents, Beliefs about science

## Abstract

On a daily basis, most people read about issues of interest from a diversity of sources. Moreover, the information they encounter frequently encompass discrepancies, ranging from minor inconsistencies to straight contradictions. Readers may construct coherent representations from discrepant contents by linking contents to their respective sources and connecting the sources with agree-disagree or other types of connectives. Across research studies, however, college-level readers' attention to sources has been found to vary according to individual, text and task dimensions. The present study tested the assumption that readers' strategies depend both on the discrepancy of the information and on the context in which the task is framed. Moreover, beliefs about science were included as potential moderator of context effects. One hundred and sixty university students were tasked to read about a series of social-scientific issues. The task was framed in either a university context or a personal context scenario. For each topic, the participants read two short texts which provided either consistent or discrepant information, and then they wrote a short overview essay. The university context had a significant impact on indicators related to a documents model representation (e.g., text switches, number of adversative connectors in the essay) and standards for presentation (e.g., time on the essay/task page, formal features of the essay). The data support a context-dependent view of reading comprehension, whereby both reading behavior and outcomes are primarily a function of the standards and goals set by the reader.

**Supplementary Information:**

The online version contains supplementary material available at 10.1007/s11145-022-10321-2.

## Introduction

Reading occurs in a broad variety of contexts and for a wide range of purposes (Snow & the RAND Reading Study Group, 2002). For example, as the Covid-19 pandemic expanded worldwide, laypersons read all sorts of texts and messages in order to answer questions such as “where did the virus originate?", to understand social distancing recommendations and to decide how strictly to comply (if at all), to find out whether they should consider vaccination, and so forth. Each of these purposes comes with relevant decisions regarding what to read and how to read it (Britt et al., 2018), potentially resulting in very different reading behaviors and outcomes. Yet, how readers represent their own reading purposes in different contexts and how they turn these purposes into goals and strategies remains surprisingly under-documented (although see Lorch et al., 1993).

In the present study, we build on a discrepancy paradigm (Braasch et al., 2012) to explore how students deal with discrepant information in different contexts when reading multiple documents. This paradigm presents readers with two discrepant claims attributed to different sources within a single text (e.g., “The detective claims that the fire in the building was due to sabotage.” vs. “The journalist asserts that the fire was caused by an electrical malfunction.”) (e.g., Braasch et al., 2012; Kammerer & Gerjets, 2014). We used this paradigm to examine how discrepant perspectives across two texts, as compared to consistent perspectives, might be addressed based on different norms or standards activated in a university context versus a personal reading context.

In the following sections, we present the extant literature concerning how different contexts affect reading, focusing on the RESOLV (REading as problem SOLVing) model of purposeful reading (Britt et al., 2018). Then we review research to date on how readers deal with discrepant information in multiple documents. Last, but not least, we use the RESOLV model to generate predictions regarding the potential influence of different contexts and of readers’ beliefs about science on dealing with intertextual discrepancies.

## How context and purposes inform reading strategies

Reading contexts are often associated with different purposes (McCrudden & Schraw, 2007). For example, a student could read multiple documents as part of a university seminar for which they have to write a summary. Or this same student could read the same documents because they are personally interested in the topic. McCrudden and Schraw (2007, p. 126) define reading purpose instructions as “prompt[ing] readers to engage in reading behaviors (e.g., inference patterns) *based on the reading context* [emphasis added].” Several studies have found that the task (as communicated through instructions or prompts) matters to how readers read and what they produce from that reading experience (e.g., McCrudden et al., 2010; Schraw et al., 1993; Wiley & Voss, 1999). For example, prompting students to produce an argument from multiple documents leads to better essays compared to writing a summary (Wiley & Voss, 1999), although the effect may be conditional upon readers' sufficient prior knowledge (Gil et al., 2010).

Whereas several studies have shown that explicit task instructions can affect reading, fewer studies have manipulated the context or situation to examine whether this more indirect manipulation can influence reading behavior, memory, or essays produced. Most of the research on reading contexts has contrasted reading to study versus reading for entertainment (e.g., Van den Broek et al., 2001). In a series of experiments, reading as if to prepare for an exam led to more coherence-building inferencing (Bohn-Gettler & Kendeou, 2014; Linderholm & van den Broek, 2002; Narvaez et al., 1999; Van den Broek et al., 2001) and better memory for the text (Bohn-Gettler & Kendeou, 2014; Van den Broek et al., 2001) compared to reading for entertainment. Van den Broek (e.g., 2001) argued that the reading context affects readers' standards for coherence, in this case, creating inferences to ensure that their representation of the expository text is coherent and complete. To our knowledge, only one study (Latini et al., 2019) investigated this context effect with multiple documents. Latini et al. (2019) observed that undergraduates spent longer reading for an exam, compared to reading for pleasure, but only when the texts were presented digitally. In contrast, they wrote longer essays in the reading for the exam condition compared to reading for pleasure condition, but only when the texts were printed out.

The psychological mechanisms underlying the context effects have yet to be uncovered. The RESOLV model (Britt et al., 2018) suggests that readers attend to cues in their environment and form a general representation of the physical and social context (called a context model). Readers use this representation to then determine their goal(s) and select strategies for achieving those goals (i.e., their task model) and regulate reading decisions. RESOLV differentiates several aspects of the physical and social context: the request itself (i.e., the task), the requester, the audience, support and obstacles, and the self. Britt et al. (2018) provide further aspects for each of these categories and example scenarios (see also Table 1 for the scenarios used in the present study).

**Table 1:** Overview over aspects of the manipulation of the context

	University context	Personal context
Scenario	University seminar	Create own podcast
Request		
Question	Write an overview	Write an overview
Criteria	Academic standards	No information/own criteria
Stakes, consequences	Need to make a good impression	–
Purpose	For a research project	As a reminder for oneself
Constraints	No other resources than system provided	
Task duration	About 1.5 h	
Requester		
Role	Professor	Best friend
Audience		
Role	Professor	Self; later: podcast listeners
Support and obstacles		
Setting/place	At home like working for university	At home like looking up information for personal reasons
Experimental setting	Formal speech (German “Sie”) Using words such as “science”, “scientific”	Informal speech (German “du”) Avoiding words such as “science”, “scientific”
Materials	Provided documents	

Studies conducted so far have compared conditions that differed on most of these dimensions of context. Thus, it is unclear so far what specific dimensions (or combination thereof) actually influence reading.

## Challenge of multiple documents: how to deal with discrepant information

Comprehending multiple documents about a topic of interest poses additional challenges to readers, compared with the reading of a single text. Documents may corroborate, complement, but also contradict each other (Britt & Rouet, 2012). When the information is discrepant, readers are prevented from creating a single, coherent mental model of the situation. In this case, creating a *documents model* can help (e.g., Britt & Rouet, 2012; Britt et al., 1999). In a documents model, readers represent the sources as well as the contents of the documents. A documents model includes an integrated mental model of the situation, portions of which are connected with the intertext model, a representation of information about the sources (e.g., author name and credentials, publication information). That is, a documents model consists of an integration of content, a representation of sources, and source-content links (i.e., who said what). If readers of multiple documents do not integrate the contents of the documents into one situation model, a *separate representations model* originates. In this case, one mental representation per document is formed. On the other side of the spectrum, when readers do integrate the contents but do not connect content to its source and do not represent sources, a *mush model* is developed. In this case, content is integrated (and even reconciled) without considering differences of sources (e.g., trustworthiness, perspective,…). Only the documents model can coherently represent discrepant information without selecting a side.

Braasch et al. (2012) tested the effect of textual discrepancies on readers’ attention to source information (i.e., who said what). They presented undergraduates with several short “news reports” and manipulated whether two sources of statements within the news report disagreed (discrepant condition) or agreed (consistent condition) about an important fact or cause. Discrepant reports resulted in more revisits to the source information and longer gaze time on the sources than consistent reports. Participants were also more likely to cite the sources in their summaries of discrepant reports. Finally, the discrepant reports resulted in better memory for the sources. These results supported the “discrepancy-induced source comprehension” hypothesis (or D-ISC), that is the view that discrepancy encourages the construction of a documents model representation by using sources to create a coherent model of the content. The D-ISC effect has been successfully replicated using this same single-text-two-source paradigm (e.g., Rouet et al., 2016; Saux et al., 2018).

Although many studies have investigated how documents models are created from discrepant multiple documents (e.g., Kammerer et al., 2016), only three experiments have used the discrepancy paradigm to test the D-ISC effect by manipulating discrepancy versus consistency across multiple texts. Two of these studies only used a single topic: Barzilai and Eshet-Alkalai (2015) had undergraduates write an argument for or against seawater desalination based on a set of documents and found better source memory in the discrepant compared to the consistent condition. Kammerer et al. (2016) had undergraduates make a recommendation for or against the use of a performance-enhancing nutritional supplement. Participants in the discrepant condition spent longer reading the source information, mentioned it more in think aloud and the essays, and evaluated a “biased” webpage more critically. However, that last point is a bit problematic because the discrepancy manipulation was confounded with corroboration of information.

To our knowledge, only one study used multiple topics and a within-subjects design in the context of D-ISC: List et al. (2021) manipulated both the discrepancy of texts and the type of reasons the texts provided for their claims (distinct vs. overlapping) in a within-subjects design such that each participant worked on four topics. They found that university students were better able to form evidentiary (e.g., referring to the mode of data collection) and thematic (e.g., whether main ideas were consistent or discrepant) connections across consistent than discrepant texts. Supplemental analyses showed that students especially had problems linking discrepant main ideas appropriately by using adversative connectors (e.g., “however”). For contextual connections (e.g., referring to the source of documents), discrepant texts were beneficial. There was no effect on sourcing and no effect of the type of reasons.

This review makes it clear that two extensions to the D-ISC effect are needed for better understanding how documents models are triggered by discrepancy in a multiple document situation. First, a stronger test of the D-ISC effect in reading multiple documents would use more than one topic, manipulated within participants, with several items, which has not been done. This design is not typically used in the multiple documents literature but with short enough texts, multiple topics can be used which allows the results to be generalized across topics and does not require a prior knowledge assessment of each topic. Second, the reading context may have an effect on the extent to which a discrepancy is needed to trigger the construction of a documents model and corresponding coherence-building inferences. Several of the studies on the D-ISC effect created a context that was more of a personal context and therefore left it to the individual to apply their own standards. Even those that did present a purpose (e.g., working for a “news agency”), did not directly compare contexts. In these cases, the discrepancy increased attention to sources. It may be, however, that an academic context will activate standards for the task model to create a complete documents model even without a discrepancy, because this is part of academic standards. Thus, we will refer to *standards of a documents model representation* as creating a representation of the task to include creating a documents model when reading multiple documents. This standard will be partially triggered by having *academic standards and norms* which are the expectations for learning and products at the university, such as considering several perspectives and giving credit to others’ work by citation.

## Beliefs about science as potential moderators of context effects

Since reading multiple potentially discrepant texts is a complex task, interindividual differences are to be expected (i.e., the "self" dimension of the RESOLV model, Britt et al., 2018). One interindividual difference variable that might play a role when reading about science-related content is readers’ beliefs about science. In the present study, we focus on two belief constructs: trust in science and the perceived utility of science. Whereas the perceived utility of science is the value that is ascribed to science for reaching one’s goals (e.g., Eccles & Wigfield, 2002; Schoor & Schütz, 2021) trust in science rather covers aspects of whether information provided by science and scientists are trustworthy (see Schoor & Schütz, 2021).

At first sight, beliefs about science sound similar to epistemic beliefs (see Schoor, 2022), that is beliefs about the nature of knowledge and knowing (e.g., Hofer & Pintrich, 1997). However, current conceptualizations of epistemic beliefs differ from beliefs about science. In dimensional models (Greene et al., 2008), epistemic beliefs are differentiated into dimensions such as certainty of knowledge, simplicity of knowledge, and justification of knowledge (e.g., by authority, by multiple sources, or by personal opinion) (e.g., Ferguson et al., 2013; Hofer & Pintrich, 1997). Although knowledge can be generated and justified by scientific inquiry, current conceptualizations of epistemic beliefs do not explicitly include it (see Schoor, 2022). Consequently, epistemic beliefs correlate only moderately with perceived utility and trust in science (Schoor, 2022).

While the relationship of epistemic beliefs with multiple document comprehension has been widely researched (e.g., Barzilai & Eshet-Alkalai, 2015; Bråten & Strømsø, 2010; Mahlow et al., 2022), beliefs about science have been neglected. We argue that multiple document comprehension often involves reading about science-related topics. In these contexts, beliefs about science may be relevant. For example, perceived utility of science is assumed to be a direct predictor of the usage of scientifically based information for own decisions (researched mainly in teacher education: e.g., Kiemer & Kollar, 2021). Beliefs about science may influence whether or not scientific sources are valued. If the quality of a source does not make a difference (i.e., in the case of low perceived utility and/or low trust in science), paying attention to sources (i.e., sourcing) is not necessary. Sourcing may become especially relevant when it serves to differentiate scientific expertise sources from sources with less expertise.

In addition, we assume that beliefs about science moderate potential context effects when reading about science. There are two competing hypotheses for how beliefs about science might moderate the context effects in this study. On the one hand, participants who place high trust in science (Nadelson et al., 2014) and consider science useful (e.g., Schoor & Schütz, 2021) would have academic standards available and be willing to use them in a task. That is they would pay more attention to sources and to creating a documents model in general. In this case, positive beliefs about science (i.e., high trust and high perceived usefulness) will have a stronger impact in the academic context. Alternatively, university students may be able to apply academic standards when a context demands it, regardless of their beliefs about science. Differences in beliefs about science may have more impact when students read in a personal context, with more freedom to decide which standards to apply. That is, in a personal context, students with positive beliefs about science would freely choose to follow the same standards as in an academic context, because they believe in these standards, whereas students with less positive beliefs in a personal context have the freedom to not follow academic norms and thus will less likely do so.

## The present study

The present study investigated the effects of context on university students' reading of multiple documents. We manipulated context in terms of expected standards while holding constant the demands of the task: reading texts to write an overview. That is we held constant the wording of the task instruction itself. Yet, we expected students to interpret this task differently in different contexts. Half of the participants received the task in a university context (i.e., as a request from their professor for a university seminar) while the other half completed the task in a personal context (i.e., to create a podcast about ideas provided by their best friend via WhatsApp, see also Table 1). To examine whether variations in context could affect readers' processing of discrepant information, for each participant, half of topic sets involved a discrepancy across the two texts and half did not. We expected two sets of variables to be impacted by the context: indicators for a standard of a documents model and indicators for standard of presentation. Moreover, we expected beliefs about science to be especially important in a personal context. This will be detailed below.

### Predictions regarding the effects on standards of a documents model representation

Following previous research, we considered several variables to be indicators for standards of a documents model representation: time on texts (e.g., Hahnel et al., 2019), text switches as behavioral indicator of corroboration (e.g., Hahnel et al., 2019), corroboration in the essay (e.g., Rouet et al., 1996; Wineburg, 1991), source citation in the essay (e.g., Braasch et al., 2012), number of adversative connectors in the essay (List et al., 2021), and whether discrepancies were explicitly addressed in the essay (Stadtler et al., 2013). We articulated several predictions regarding readers' standards for constructing a documents model representation:

#### DIS-C effect

First, we expected the DIS-C effect to be replicated in our study. As formulated in the D-ISC hypothesis (Braasch et al., 2012), discrepant texts should raise the attention to sources and encourage the construction of a documents model representation, because a documents model allows discrepant information to be represented in a coherent way by tagging content with its source (e.g., Braasch et al., 2012; Rouet et al., 2016; Saux et al., 2018). Thus, we expected discrepant (as compared to consistent) topics to overall lead to a better documents model representation, which is supposed to be visible in the above-mentioned indicators:

H1 (Standard of a documents model representation – D-ISC effect): A discrepancy across documents results in longer time on texts, more switches across texts, and more corroboration in the essays, more citations of sources and a higher number of adversative connectors in the essays.

#### Context effect on standards of a documents model representation

We expected the reading context to shape the importance of academic standards that students apply during work on the texts (Linderholm & van den Broek, 2002; Narvaez et al., 1999; Van den Broek et al., 2001; see Schoor & Bannert, 2013). A university context (as compared to a personal context) should activate academic norms and raise standards for creating a documents model for one’s own understanding as well as reflecting this information in their overview for their audience. In contrast, the personal context should activate one’s personal norms. The reader may not set the expectation that a documents model is required but instead assume that selection of a side is more important for this context. We expected that this would be reflected in the above-mentioned indicators of standards of a documents model representation:

H2 (Standard of a documents model representation– context effect): The university context should lead to a longer reading time, more text switches, more corroboration in the essays, more source citation, a higher number of adversative connectors, and more explicit addressing of a discrepancy than a personal context.

#### Interaction effects of context and discrepancy on standards of a documents model representation

With regard to possible interactions of context with the discrepancy manipulation, several competing predictions may be put forward. On the one hand, in the university context, in which academic norms are expected, sources might be deemed so important that they are always attended, regardless of the presence of a discrepancy between the texts (i.e., a ceiling effect). This would mean that a D-ISC effect (e.g., Braasch et al., 2012; Rouet et al., 2016; Saux et al., 2018) would not be visible in the university context, but only in a personal context in which academic norms are dependent on individual standards (i.e., the "self" dimension of the RESOLV model, Britt et al., 2018, e.g., beliefs about science, see Schoor & Schütz, 2021). Alternatively, a discrepancy effect may only be apparent in an academic context, that is when students use a higher standard of coherence. In this interpretation, discrepant information and sources may not be deemed important in a personal context, so that no DIS-C effect occurs. Of course, a third alternative would be simple additive effects of discrepancy and context (i.e. no interaction effect). So far, neither RESOLV (Britt et al., 2018) nor previous empirical studies favor one of these alternatives. Thus, the following alternative hypotheses were formulated:

H3a (Standard of a documents model representation—interaction of context and discrepancy: ceiling DIS-C effect in university context): There is an interaction effect of context and discrepancy such that the DIS-C effect (see H1) only occurs in the personal context but not in the university context.

H3b (Standard of a documents model representation—interaction of context and discrepancy: irrelevance of sources in personal context): There is an interaction effect of context and discrepancy such that the DIS-C effect (see H1) only occurs in the university context but not in the personal context.

H3c: There is no interaction effect of context and discrepancy.

#### Effects of beliefs about science on standards of a documents model representation

When reading about science-related topics, we assumed beliefs about science (e.g., Schoor & Schütz, 2021) – in analogy to epistemic beliefs (e.g., Barzilai & Eshet-Alkalai, 2015; Bråten & Strømsø, 2010; Mahlow et al., 2022)—have an influence on how well students process multiple documents. More specifically, we expected that students with more positive beliefs about science would develop a better comprehension of multiple documents about a science-related topic, that is to develop a documents model representation. Thus, we expected more positive beliefs to be related to higher values on indicators for the standards of a documents model representation:

H4a (Standard of a documents model representation—beliefs about science): Beliefs about science are positively related to indicators for the standards of a documents model representation.

In addition, we expected beliefs about science to interact with the context. As detailed above, more positive beliefs about science standards (as part of the "self" dimension of the RESOLV model, Britt et al., 2018) may have a greater impact on the standards of a documents model in the university context, because students with positive beliefs have academic norms available and are motivated to use them. As a competing hypothesis, more positive beliefs about science may especially impact the standards in a personal context in which students are free to act according to their own norms and values. In this case, these values would be reflected in beliefs about science. According to RESOLV (Britt et al., 2018) both types of interaction are plausible, which is why the following two alternative predictions were put forward:

H4b (Standard of a documents model representation—interaction of context and beliefs about science: norm availability): Positive beliefs about science have a greater impact on indicators for the standards of a documents model representation in the university context than in the personal context.

H4c (Standard of a documents model representation—interaction of context and beliefs about science: own norms): Positive beliefs about science have a greater impact on indicators for the standards of a documents model representation in the personal context than in the university context.

With regard to possible interaction effects of beliefs about science and discrepancy, and consequentially also with regard to a possible three-way interaction, there has not been enough prior work to come up with reasonable hypotheses.

### Predictions regarding the effects on standards of presentation

In addition to effects of the context on the standards of a documents model representation, we expected that the importance of academic standards activated by a university context would also be visible in standards of presentation. That is, in addition to the content that is considered in the standards of a documents model representation (see section “Predictions regarding the effects on standards of a documents model representation”), we also considered a more formal or superficial level of the essays. We expected that in a university context, the task to “write an overview” would interpreted differently than in a personal context, as predicted in RESOLV (Britt et al., 2018), also with regard to how this overview was supposed to look like.

The university context should require a more polished product because the product will be evaluated by an authoritative third-party (i.e., a professor) and it is important to make a good impression (see Stoeber & Hotham, 2013), whereas in a personal context, when writing an overview for oneself, simple notes may be enough, because nobody needs to be impressed. Thus, it was expected that an academic context should result in a higher standard for a polished product or presentation (see Cho & Choi, 2018). As indicators for standards of presentation, the time spent on writing the essay, the length of the essay, and whether or not the essay was a continuous text were considered.

H5 (Standard of presentation—context effect): In a university context, students spend more time on the essay page and write longer and more polished essays (as evidenced in participants writing more cohesive texts as opposed to mere sets of notes).

With regard to discrepancy, we had no hypotheses for the standards of presentation. With regard to beliefs about science, we exploratorily researched whether they had a main or interaction (with context) effect on the standards of presentation, since we assumed that they could activate appropriate standards in a similar way as for the standards of a documents model representation (see explanation for H4a and H4b).

## Method

### Sample

Participants were 160 university students of a German university mainly enrolled in sociology (26.3%), educational science (16.9%), psychology (16.3%), or teacher education (13.8%). They were 18 to 32 years old (*M* = 23.6, *SD* = 3.02, 76.9% female) and on average in their 8th semester at the university (*M* = 7.9, Median = 8, *SD* = 4.11, range 1–18). They were recruited via university mailing lists, online courses, and social media groups related to the university. They received 25 € as an incentive.

The sample size was determined based on a power analysis (with GPower 3.1: Faul et al., 2009) and considerations about feasibility and cost. With the present sample size, a small to medium effect (f = 0.15) in the within-between interaction (interaction of context and discrepancy) can be detected with a power of 1 − β = 0.95 and α = 0.05. A medium between-effect (f = 0.25, main effect of context) can be detected with a power of 1 − β = 0.80 and α = 0.05.

### Design

As independent variables, *context* was manipulated as a between-subject variable, and *discrepancy* was manipulated as a within-subject variable. The participants were either introduced into a university scenario or in a personal scenario (context manipulation). Within this scenario, they worked on 8 target plus 6 filler topics. The target topics were manipulated to convey either consistent or discrepant information across two texts (discrepancy manipulation). In addition, the order of target topics, the discrepancy of each target topic (i.e., whether a given topic was presented in consistent or discrepant manner), and the order of the two texts within the target topics were balanced across participants.

### Procedure

Due to the 2020 pandemic situation, the study was conducted online via a web browser. The participants were randomly assigned to a university or personal context condition. On the first page of the material, the participants received information about the study and gave their informed consent (in accordance with APA human subjects principles). Then, demographics and prior beliefs about the topics were assessed, before the context manipulation was introduced. In order to make the participants thoroughly memorize their scenario (i.e., the context manipulation), they were told that they would be asked to recall the scenario on the next page without the possibility to go back, which is what happened. That is, their context and task model were assessed with open-ended questions (not analyzed in the present study). After this, they had the opportunity to take a short break, before the work on the topics began. They had to work on 14 topics, 8 of which were target topics. Although using several different topics is uncommon in the literature, it is not without precedent (e.g., Bråten & Strømsø, 2003; List et al., 2021; Rouet et al., 1996, 1997). We chose this procedure to avoid topic effects, for example due to interest in the topic or interestingness of the texts. The first two topics the participants worked on were filler topics, so that they would serve as practice topics. The other filler topics were randomly mixed in between the target topics to conceal the true intention of the study. Therefore, filler topics could have only one text or even no text (in this case, participants were supposed to write an essay about the topic without available texts) or two texts with very different sources with regard to trustworthiness. The intention was to conceal that we were interested in how students deal with discrepant and consistent texts in which sources are of equal trustworthiness and thus a discrepancy cannot be decided by ignoring the text of an untrustworthy source. After each topic, the participants had the opportunity to take a short break. After all 14 topics, they had to fill in the post-test measures on the target topics, that is on their memory for texts, sources, and discrepancies.^1^ Then, several manipulation control measures (i.e., did they behave as in a real situation, second context and task model assessment, text comprehensibility ratings) as well as beliefs about science were assessed. The participants spent on average about 2.5 h on the study. The study was part of a larger project (i.e., a series of studies), which had been approved by the local ethics committee.

### Material and instruments

#### Context manipulation

The manipulation of context was done in a way to increase the salience and importance of academic norms in the university context, while emphasizing the freedom to work based on own personal norms in the personal context. A prerequisite was that the actual task could be held constant across context, and that based on this task an outcome (i.e., a text written by the participants) could be created. See Table 1 for an overview over all aspects of the context manipulation. In the following, we describe the most important aspects of manipulation. In parentheses, we provide the aspect according to RESOLV and as used in Table 1. The complete original and translated instructions for the context manipulation are available via the Open Science Framework (OSF) with the 10.17605/OSF.IO/YSJGH.

**Fig. 1:**
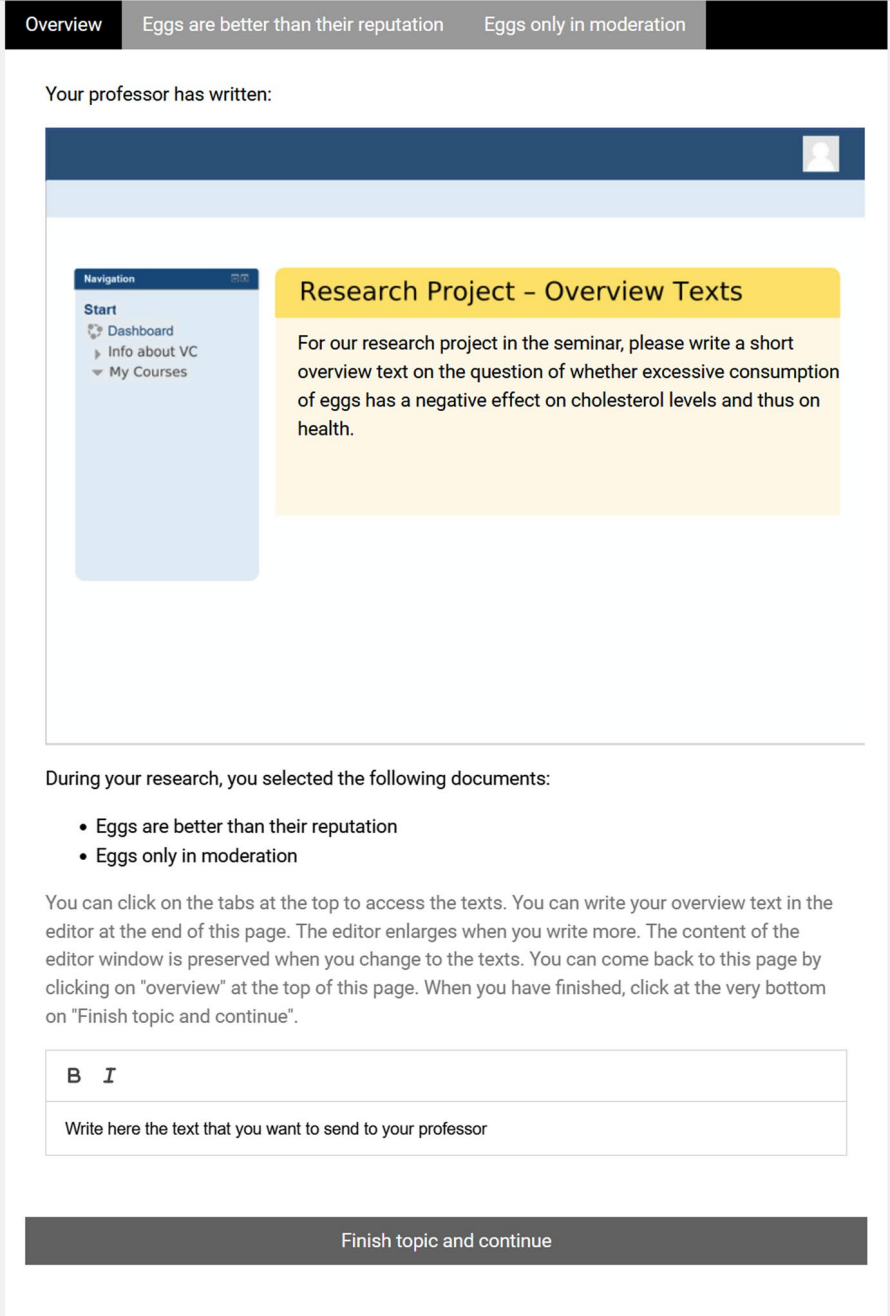
Computer environment in the university context

In the university condition, the participants were supposed to imagine that they attended a university seminar (*scenario*) of the professor with who they want to write their final thesis. They wanted to make a good impression so that the professor would accept them for this thesis (*stakes, consequences*). Within the seminar, their task was to write overview texts (*question*) over several generally understandable topics for a research project (*purpose*). Their professors would read the overview texts they wrote (*audience*). They already had searched for and selected texts they wanted to read (*materials*) and today they wrote the overview texts. The computer environment mimicked the appearance of the learning management system of the university (see Fig. 1).

**Fig. 2:**
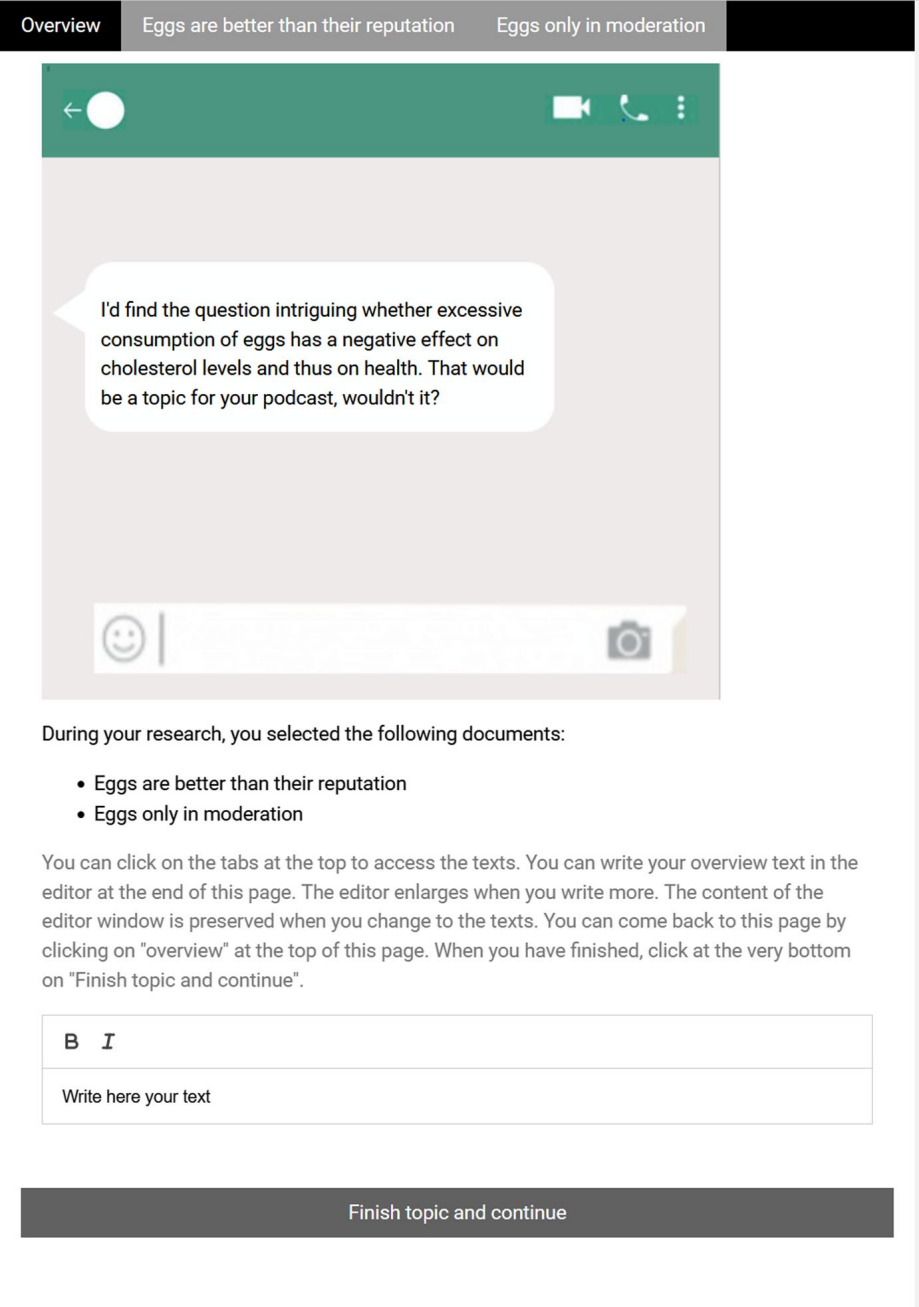
Computer environment in the personal context

In the personal condition, the participants were told to imagine that they wanted to create their own podcast (*scenario*). That they had help for the technical issues and that now they would think about potential topics for the podcast. Their best friend (*requester*) had sent them suggestions for topics, they already had searched for and selected texts they wanted to read (*materials*) and today they wrote overview texts (*question*) for themselves (*audience*) as a reminder (*purpose*). The computer environment mimicked a WhatsApp conversation (see Fig. 2).

In order to additionally increase the salience of academic norms in the university condition or to decrease the real scientific context of the study in the personal condition, several other measures were taken with regard to language used (*experimental setting*) and self-arranged environmental setting (*setting/place*).

#### Manipulation control question

At the end of the procedure, the participants were asked to report on a 5-point Likert scale whether they had behaved in the experiment as they would have in the real situation. The answer options were *never*, *most of the time not*, *now and then*, *most of the time*, and *all the time*.

#### Texts and discrepancy manipulation

In both context conditions, the topics (from the domains of health, sports, or education and potentially of interest to the general public) were introduced by a question (e.g., “Are sweeteners such as Aspartame in the amounts contained in food harmless to health?”) that was posed either by the professor or the best friend, depending on the scenario (see Figs. 1, 2). In all target topics, the participants were provided with two texts on the topic (160–180 words, medium readability LIX: Lenhard & Lenhard, 2014; readability was kept very similar across texts within a topic, i.e. in a range of approximately 10 points of the LIX) that were either discrepant or complementary with regard to the main claim and the provided reasons (see Table 2).

**Table 2:** Design of consistent and discrepant texts

Text11	Text12	Text21	Text22
Claim: pro	Claim: pro	Claim: contra	Claim: contra
Reason 1 pro Reason 2 pro		Reason 1 con Reason 2 con	
	Reason 3 pro Reason 4 pro		Reason 3 con Reason 4 con

Each participant received only two of these texts. In the consistent condition, participants received either text11 and text12, or text21 and text22. In the discrepant condition, participants received either text11 and text21, or text12 and text22. It was balanced across participants which of the two possible combinations of texts per condition they received

Each text was provided on one screen, showing the title of the text, the source (i.e., the name and profession of the alleged author, who was always an expert for the topic), and the content. Each text content started with a short introduction into the topic, followed by the main claim of the text. Each text provided two distinct reasons for its main claim (see List et al., 2021) and additional explanatory and supporting information. In contrast to the study by List et al. (2021), the supporting reasons were distinct across texts in the consistent condition and exactly opposite (e.g., by introducing the word “not” or by using an antonym) in the discrepant condition.

#### Computer environment

After the (context-dependent) instructions, participants started working on the topics. For each topic, they first landed on the task/essay page (displayed in Figs. 1, 2) which introduced the question of the topic, depending on the context condition in a learning management style or as WhatsApp communication (see upper half of Figs. 1, 2). Below the learning management/WhatsApp image with the question, we provided the titles of the documents that they had allegedly selected for reading and that were provided within the study. They could access these texts by using the navigation in the top-most line of the browser window, which provided the title of the texts as links as well as a link back to the task/essay page (“Overview”). The lower part of the task/essay page consisted of a short explanation of how to use the system, and the editor itself, which was enlarged when more text was written. Through this layout of the computer environment, we were able to log time on each page (i.e., task/essay page, text 1, text 2) as well as all switches between them.

#### Behavior: time data and text switches

The following indicators were built using the R package LogFSM (Kroehne, 2020; Kroehne & Goldhammer, 2018). For each target topic, *time on texts* was created by summing the time that the participants spent on text pages (i.e., both first reading and repeated reading) and *time on task/essay page* was created by summing the time spent on the task page which presented the task and provided space for writing the essay.^2^ If they never opened the page, their score was set to zero. The time was measured on the millisecond level. Some text page visits were unrealistically long. Since this was an unsupervised online study and participants might have been interrupted or took an unplanned break, text page visits that took longer than 4.5 min were excluded for calculating the time spent on the texts.^3^ This applied to 58 out of 11,538 text page visits (0.5%).

The *number of text switches* was used as an indicator of the degree to which participants compared content. A text switch was counted when participants visited a text either from another text (e.g., Text2 from Text1 or Text1 from Text2) or by way of the task page (Text1—task page—Text2 or Text2—task page—Text1). The frequency of these sequences was summed up per participant and topic. Because simply reading all the texts requires at least one such sequence, the count was subtracted by 1. In addition, for those participants who did not revisit the texts of the topic, the sum of text switches was set to 0. Thus, this indicator represents comparison processes above a single visit of each text.

#### Use of information: coding of essays

The essays that the participants wrote for each topic were coded with regard to several aspects by two independent trained student raters. For the standard for presentation, *length* was defined as the number of words counted using Microsoft Word. As *continuous essay* it was coded dichotomously whether the essay was presented as a continuous essay (= 1) or a list of points (= 0; Cohen’s κ = 0.93). For the standards of a documents model, source citation, numbers of adversative connectors, corroboration, and explicitly addressing the discrepancy were considered. *Source citation* was a dichotomous coding of whether or not the content for at least one of the texts included a mention of one or more detailed source elements such as name or profession (on the basis of Braasch et al., 2012; Bråten et al., 2015) (Cohen’s κ = 0.95). The *number of adversative connectors* was counted by an R script based on list of the Leibniz Institute of German Language (https://grammis.ids-mannheim.de/konnektoren?lemma=&synclasses=&synconnect=0&positions=&posconnect=0&semclasses%5B0%5D=adversativ&semconnect=0&page=2). *Corroboration* was a dichotomous indicator of whether or not in the essay at least one of the expressions displayed in Table 3 was used (determined by an R script), thus providing indirect evidence that students have been corroborating (see Rouet et al., 1997; Wineburg, 1991). *Explicitly addressing the discrepancy* was a dichotomous coding (for the discrepant topics only) of whether the student mentioned in the essay that the topic was discrepant, based on Stadtler et al. (2013) (Cohen’s κ = 0.84).

**Table 3:** Expressions indicating corroboration in essays (German original and English translation)

German	English
“beide Texte”, “beiden Texten”	Both texts
“beide Quellen”, “beiden Quellen”	Both sources
“beide Autoren”	Both authors
“beide Artikel”, “beiden Artikeln”	Both articles
“andere Text”, “anderen Text”	Other text
“andere Quelle”, “anderen Quelle”	Other source
“andere Autor”, “anderen Autor”	Other author
“andere Artikel”, “anderen Artikel”	Other article
“gleiche Meinung”, “gleicher Meinung”, “gleichen Meinung”	Same opinion
“anderer Meinung”, “andere Meinung”, “anderen Meinung”	Different opinion
“gleiche Position”, “gleichen Position”	Same position
"andere Position", "anderen Position", "anderer Position"	Different position
“einig”	Agree
“widersprechen”, “widerspricht”	Contradict
“widersprüchlich”	Contradictory

German expressions take different possible grammatical structures into account

#### Beliefs about science

As beliefs about science, both the perceived utility of science and trust in science were assessed. The perceived utility questionnaire (Schoor & Schütz, 2021) covers two scales: the utility of science and the utility of personal experiences. Each scale comprised 4 items that were answered on a 5-point Likert scale. A confirmatory factor analysis (CFA) showed an acceptable fit in the present study (χ^2^ = 38.70, *df* = 19, *p* = 0.005; RMSEA = 0.08; CFI = 0.94; SRMR = 0.06). The internal consistencies of the scales were good (McDonald’s ω^4^: utility of science: 0.82; utility of personal experiences: 0.80). The two latent factors were negatively correlated (*r* = − 0.27, *p* = 0.006).

Trust in science was measured by means of a German version of Nadelson et al.’s (2014) questionnaire (Schoor & Schütz, 2021). In the present study, a CFA showed a suboptimal fit (χ^2^ = 49.49, *df* = 19, *p* < 0.001; RMSEA = 0.10; CFI = 0.92; SRMR = 0.05). However, the scale had a very good internal consistency (McDonald’s ω: .86).

For both perceived utility of science and for trust in science, the mean of items was used. For the analyses with regard to their potential main and moderating effects, this score was z standardized. The perceived utility of personal experiences scale was not used in the present study.

### Data analysis and availability

The data that support the findings of this study have been deposited in the OSF with the 10.17605/OSF.IO/XSG32. The data were analyzed by means of (generalized) linear mixed models with the R package *lmerTest* (Kuznetsova et al., 2017). For dichotomous dependent variables, the logit link function within the binomial family and the “bobyqa” optimizer were used. For all other variables, the Gaussian family was used although for some variables (count variables, time data), the normality assumption was violated. However, linear mixed-effect models are quite robust to violations of distributional assumptions and using Gaussian models is in many cases still the best way of analyzing the data (Knief & Forstmeier, 2021). Following Knief and Forstmeier (2021), we additionally recalculated the models for count and time variables after a rankit transformation (with the R package *bestNormalize*; Peterson, 2021) and report only results that stay significant. For the sake of better interpretability, we report coefficients for non-transformed data.

For analyzing H1 (DIS-C effect), the respective dependent variable was predicted by discrepancy. For H2 (context effect on standards of a documents model representation) and H5 (context effects on the standards of presentation), the respective dependent variable was predicted by context. For H3 (interaction effects of context and discrepancy), the respective dependent variable was predicted by context, discrepancy, and their interaction. For H4 (effects of beliefs about science), the respective dependent variable was predicted by context, discrepancy, their interaction, the belief (utility of science or trust in science) and, in a second model, its interaction with context. Moreover, an intercept and random effects for the participant and the topic were included. If the topic effect was (close to) zero, it was excluded to avoid estimation problems. In a baseline model including intercept, participant and topic effects, also the position of the topic was included in order to control for potential motivation loss or fatigue effects. If the effect of the position was significant, position was kept in the analyses. If it was not, position was dropped for the analyses, also because additionally including the position caused estimation problems in several models. Reference categories used to estimate the effect of categorical predictors were as follows: for context effects, the personal context; and for discrepancy effects, the consistent topics.

## Results

**Fig. 3:**
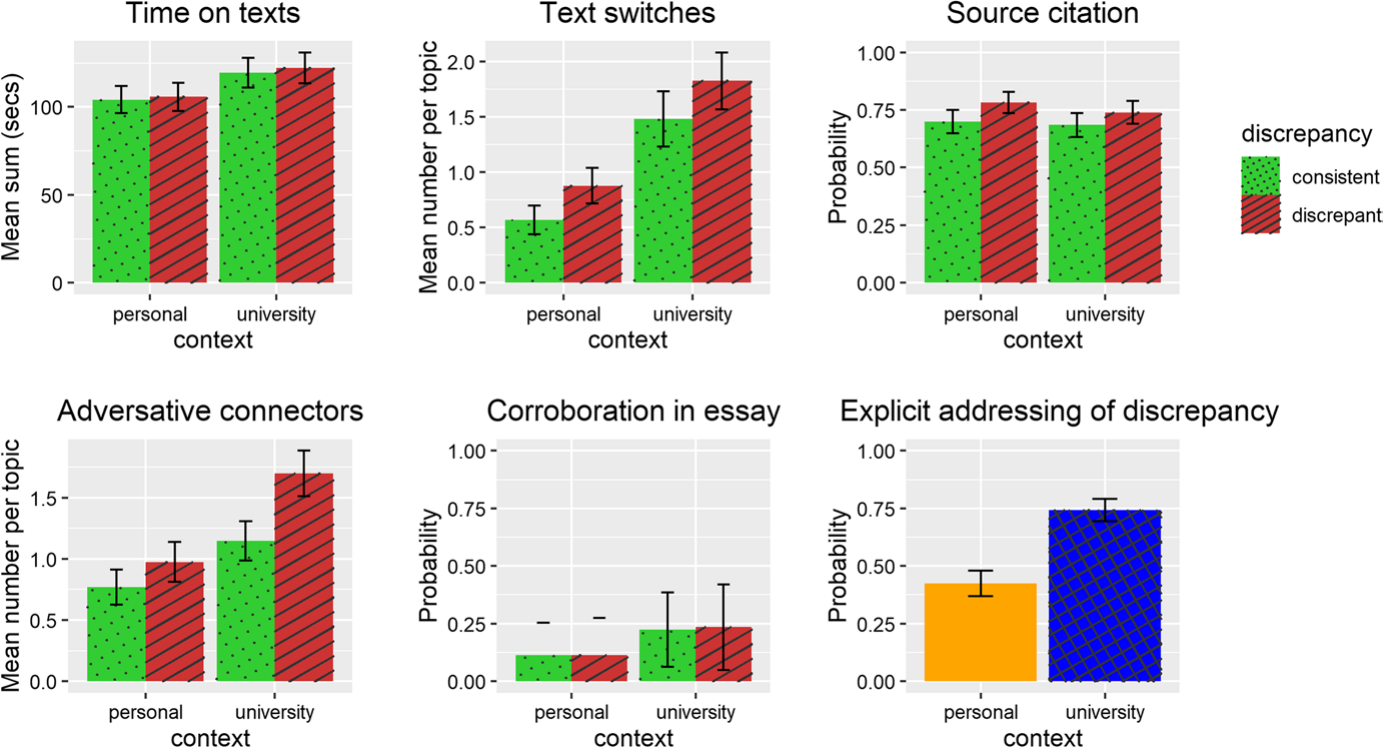
Results regarding standard of documents model. Error bars represent confidence intervals

Descriptive statistics for all measured variables and a list of significant results (including position effects) are shown in Table 4. Zero-order correlations are displayed in Table 5. The results are illustrated in Figs. 3 (standard of a documents model representation) and 4 (standard of presentation).

**Table 4:** Descriptive statistics

	Scale range	M (SD)	Personal consistent	Personal inconsistent	University consistent	University inconsistent	Results^a^
*Documents model*							
Time on texts (s)	0–∞	112.91 (75.28)	104.20 (70.00)	105.79 (72.35)	119.36 (77.05)	122.32 (79.94)	Uti*C, P
Text switches	0–∞	1.19 (1.95)	0.57 (1.19)	0.88 (1.48)	1.48 (2.28)	1.83 (2.35)	C, D, Uti, P
Source citation	0–1	0.73 (0.45)	0.70 (0.46)	0.78 (0.41)	0.68 (0.47)	0.74 (0.44)	D
Number of adversative connectors	0–∞	1.15 (1.53)	0.77 (1.29)	0.98 (1.49)	1.15 (1.47)	1.70 (1.70)	C, D, C*D, P
Corroboration in essay	0–1	0.17 (0.38)	0.11 (0.32)	0.11 (0.32)	0.22 (0.42)	0.23 (0.42)	C
Explicitly addressing the discrepancy	0–1	0.58 (0.49)	NA	0.42 (0.49)	NA	0.74 (0.44)	C, Trust*C
*Presentation*							
Time on task/essay page (s)	0–∞	313.93 (194.51)	256.40 (152.27)	275.61 (186.56)	341.48 (198.52)	382.22 (209.92)	C, D, P
Length	0–∞	96.49 (52.08)	85.92 (50.07)	94.12 (56.01)	98.31 (47.62)	107.63 (52.11)	D, P
Continuous essay	0–1	0.72 (0.45)	0.46 (0.50)	0.44 (0.50)	0.98 (0.15)	0.99 (0.11)	C, C*D

^a^Significant main and interaction effects (indicated by *) of context (C), discrepancy (D), trust in science (trust), and perceived utility of science (uti). Interactions of beliefs about science with discrepancy have not been analyzed. If there was a significant position (P) effect in the baseline model, position was controlled for in the analyses

**Table 5:** Zero-order correlations of variables

	(2)	(3)	(4)	(5)	(6)	(7)	(8)	(9)
*Documents model*								
(1) Time on texts (s)	.43***	.11***	.12***	.08**	.12**	.32***	.11***	.01
(2) Text switches		.04	.16***	.18***	.25***	.21***	.10***	.16***
(3) Source citation			.01	.10***	.29***	.20***	.29***	-.19***
(4) Number of adversative connectors				.06*	.23***	.29***	.42***	.22***
(5) Corroboration in essay					.66***	.15***	.15***	.43***
(6) Explicitly addressing the discrepancy						.25***	.21***	.50***
*Presentation*								
(7) Time on task/essay page (in secs)							.57***	.17***
(8) Length								.12***
(9) Continuous essay								

Pearson correlation for intercorrelations of continuous variables. Point-biserial correlation for intercorrelations of dichotomous with continuous variable. Tetrachoric correlation for intercorrelations between dichotomous variables. For each person, values for all eight target topics were entered where applicable. Significance testing was not corrected for repeated measurement. * p < .05; p < .01; *** p < .001

**Fig. 4:**
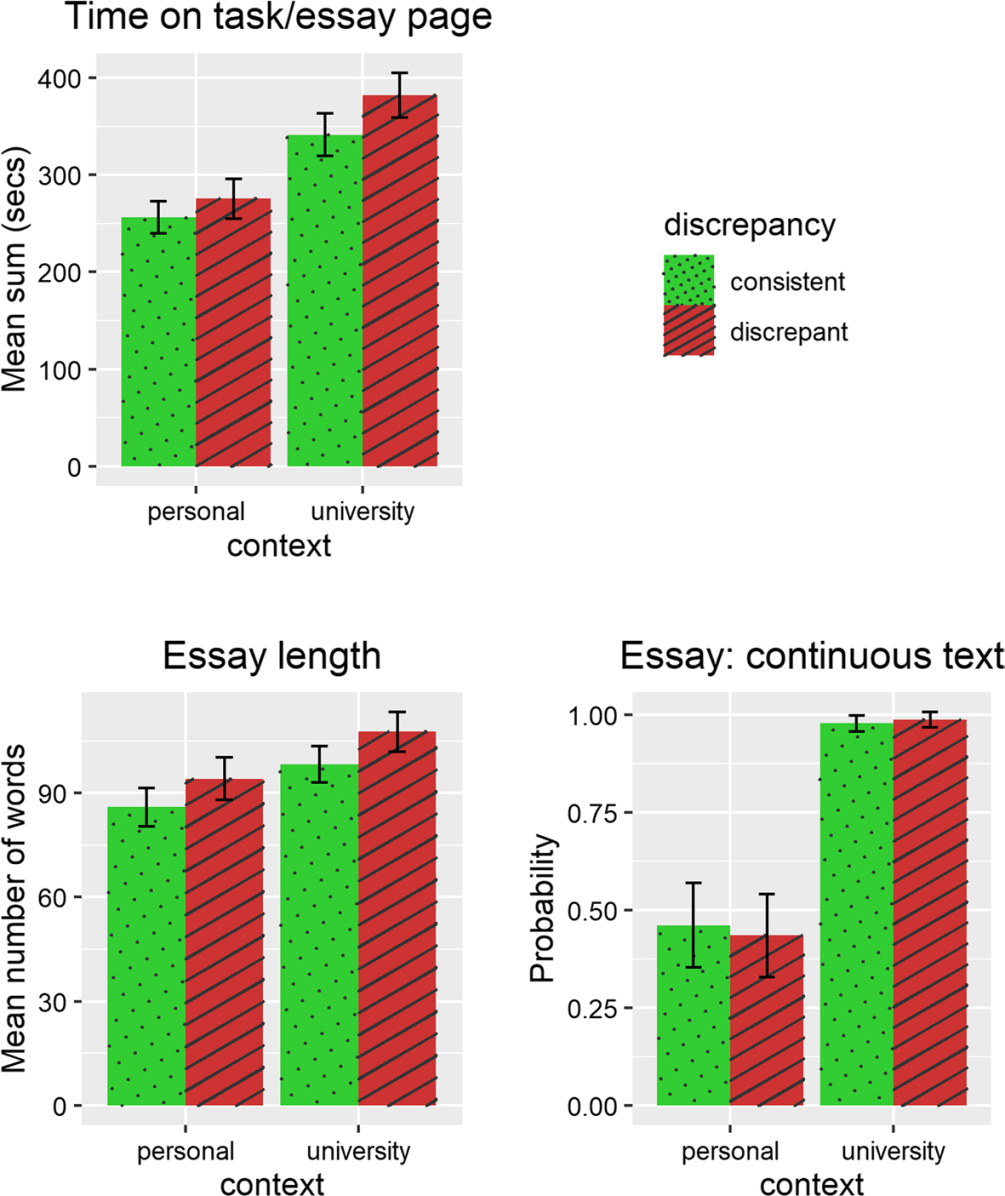
Results regarding standard of presentation. Error bars represent confidence intervals

Most of the participants (61.25%) reported in the manipulation control question that they *most of the time* or *all the time* behaved in the experiment as they would have in the real situation. Additional 30% reported that they behaved *now and then* as in the real situation. There was no significant difference across contexts (behaved as in the real situation at least *most of the time*: χ^2^ = 1.29, df = 1, *p* = 0.256; behaved as in the real situation at least *now and then*: χ^2^ = 1.96, df = 1, *p* = 0.162).

### Hypothesis 1: DIS-C effect

With regard to discrepancy effects on standards of a documents model representation, we found no effect of discrepancy on time on texts or corroboration in the essays. Yet, we found effects of discrepancy on text switches (β = 0.08, *p* < 0.001), source citation in the essay (odds ratio [*OR*] = 3.67, *p* < 0.001), and number of adversative connectors in the essay (β = 0.12, *p* < 0.001) such that in discrepant topics students more often switched texts, cited sources in their essays, and used more adversative connectors than in consistent topics. Thus, H1 (Standard of a documents model representation—D-ISC effect) could partly be confirmed.

### Hypothesis 2: context effect on standards of a documents model representation

With regard to context effects on standards of a documents model representation, we found no effect of context on time on texts or on source citation, but we did find effects of the context on the number of text switches (β = 0.24, *p* < 0.001), the number of adversative connectors in the essay (β = 0.18, *p* < 0.001), corroboration in the essay (*OR* = 3.22, *p* < 0.001), and on whether or not the discrepancy was explicitly addressed in the essay (*OR* = 19.66, *p* < 0.001). All of them were more frequent in the university context as compared to the personal context. Thus, H2 (Standard of a documents model representation– context effect) could be partly confirmed.

### Hypothesis 3: interaction effects of context and discrepancy on standards of a documents model representation

With regard to possible interaction effects of context and discrepancy on standards of a documents model, we had three competing hypotheses: A more pronounced DIS-C effect in the personal context (H3a), a more pronounced DIS-C effect in the university context (H3b) or no interaction effect at all (H3c). Although we did find an interaction effect on the number of adversative connectors (β = 0.10, *p* = 0.014) such that the discrepancy effect was larger in the university context (supporting H3b), we found no interaction effect of context and discrepancy on the time spent reading the texts, on the number of text switches, on source citation in the essays, or on corroboration in the essays. Thus, the results mainly speak in favor of the last hypothesis (H3c).

### Hypothesis 4: effects of beliefs about science on standards of a documents model representation

With regard to beliefs about science, we had expected that they would have a positive main effect on standards of a documents model (H4a), and we had two competing hypotheses how they could interact with context: that the influence of beliefs about science would be grater in the university context (H4b) or that their impact would be greater in the personal context (H4c).

With regard to main effects of beliefs about science, we found that a higher perceived utility of science was related to more text switches (β = 0.13, *p* = 0.007). There were no main effects of beliefs about science on the time spent reading the texts, source citation, number of adversative connectors, corroboration in the essay, or explicitly addressing the discrepancy (in discrepant topics), and no significant main effect of trust in science on text switches. Thus, H4a (Standard of a documents model representation –beliefs about science) mainly had to be rejected.

With regard to interaction effects, we found an interaction of trust in science and context on whether or not the discrepancy was explicitly addressed: In a university context, more trust in science made it more likely that the discrepancy was made explicit (*OR* = 3.63, *p* = 0.034). There was no interaction effect of utility of science and context on this measure. However, there was an interaction effect of utility of science with context for time on texts (β = 0.20, *p* = 0.014) such that in a university context, a higher utility of science was associated with longer time on the texts. There was no analogous effect of trust in science and context. Moreover, there were no interaction effects of beliefs about science on the number of text switches, source citation, corroboration in the essay, or the number of adversative connectors. Thus, we found only very few results supporting H4b, but mainly both H4b and H4c had to be rejected.

### Hypothesis 5: context effects on the standards of presentation

With regard to context effects on standards of presentation, we found that students spent more time on the essay page (β = 0.25, *p* < 0.001) and were much more likely to write a continuous essay as compared to single phrases and bullet points (*OR* = 7,694,331,690.17,^5^*p* < 0.001) but did not write longer essays in the university context than in the personal context. Thus, H5 was mainly confirmed.

### Effects of discrepancy, beliefs about science, and their interaction with context on standards of presentation (exploratory analysis)

The discrepancy of topics had an effect on standards of presentation: Students spent more time on the essay page (β = 0.08, *p* < 0.001) and wrote longer essays (β = 0.07, *p* < 0.001) for discrepant as compared to consistent topics. There was no effect of discrepancy on whether students wrote a continuous essay.

Discrepancy and context interacted such that students in the university condition were even more likely to write continuous essays for discrepant topics (*OR* = 6.26, *p* < 0.001). There were no interaction effects on time spent on the essay page or the length of the essays.

With regard to beliefs about science, we found neither significant main effects on indicators of standards of presentation nor interaction effects with context.

## Discussion

The present study assessed the combined effects of context and textual discrepancies on readers’ standards for a documents model representation and standards for presentation. We contrasted a university context that emphasized evaluation with regard to academic norms with a personal context that lacked this emphasis. In addition, we researched effects of beliefs about science. The context manipulation impacted readers’ standard for a documents model representation and standard of presentation. With regard to discrepancy, the experiment replicated and extended prior findings.

### Effects of the context on standards of a documents model representation and standards of presentation

The first main goal of the study was to examine context effects on participants’ standards for constructing document models, as opposed to simpler representations. In the university context, participants more frequently switched between texts, used more adversative connectors when writing about the documents, used more corroborating expressions in the essays, and were more likely to explicitly address potential discrepancies in their essays. These findings strongly suggest that participants in the university condition attached more importance to conveying a balanced presentation of the content of the documents, which required the construction of a documents model (e.g., Britt & Rouet, 2012).

With regard to standards for presentation, we found that participants in the university context spent more time on the task/essay page than participants in the personal context. This is consistent with the finding that participants in a university context almost always wrote a continuous essay, as opposed to bullet points in a personal context. Writing continuous text requires more time than just writing some notes. Thus, the participants in the university context condition invested time in communicating their findings, probably because they had a different standard for how the product had to look like, that is for the presentation. Differences in readers’ standards for presentation are probably due to the presence of an explicit audience in this context (see Cho & Choi, 2018). However, further studies are needed to clarify whether it is the audience alone that produced the effects observed in the present study.

All in all, the findings can be interpreted on a general level as support for the RESOLV model and on a more specific level with regard to differences between the university and the personal context. First, the findings on context effects support on a more general level the notion that students interpret a given task (e.g., “write an overview”) against the background of the specific context as it was suggested in RESOLV. In the present study, the actual task was the same in both conditions. We provided the students with background information about the context, but we did not instruct them to, for example, write nicely because they write for their professor. This is an inference that the participants themselves drew, only because we placed the current task in one or the other context. Thus, the present findings support the notion that task instructions are interpreted differently depending on the context of the task. As suggested in RESOLV, readers seem to use context features to infer what exactly they are supposed to do and how to understand the task. Our data support the view that contextual cues shape students' interpretation of an explicit task request (e.g., “write an overview”), and their generation of different reading goals and strategies, depending on the context.

With regard to university versus personal context, we suggest that a university context prompt the activation of academic norms and standards which are not (or not as strongly) activated in a personal context. These academic norms probably encompass not only norms about sources, source citations, and a balanced presentation of the literature, but also norms about an academic style of writing, which is connected to the use of sophisticated words, including connectors, and that mere notes are not sufficient. In contrast, in the personal context these norms are not (or not as strongly) activated. Idiosyncratic norms can be applied instead. Moreover, the personal context did not involve any external audience for the essay. To put it in RESOLV terms, the only audience was the self (Britt et al., 2018). Thus, differences in audience (and not just the academic norms) may have contributed to the observed context effect.

Yet, in the present study we could observe most of this only indirectly. That is, we did not directly measure the goals and strategies that students represented in their task model nor their norms and standards that were supposedly activated by the different contexts. Further studies should explicitly test both goals/strategies and norms/standards to confirm our interpretation of results. More work is also needed on how people interpret tasks. For example, there may be one aspect in task understanding (e.g., the standard to write continuous text in the university condition) that drives other results (e.g., the effect on the length of essay) or the context influences several aspects independently (which the low correlations in Table 5 suggest for the present study). Future studies, which may also use other methods such as think-aloud, may give further insight into this.

We also did not assess the mental representation of the texts, but only the students communication thereof. Thus, we cannot conclude that the mental representation of the texts is different across contexts. A different communication may be due to a different mental representation, but it could also be caused by a different understanding of what needs to be communicated. Since we found no context effect on the time spent reading, but an effect on the time spent on the task/essay page, this may be a first hint that the mental representation of the texts may not necessarily be different across contexts, but how a representation is presented. Yet, this needs further exploration.

### Effects of discrepancy: replication and extension of DIS-C effects

As in prior studies (e.g., Braasch et al., 2012; Kammerer & Gerjets, 2014; Kammerer et al., 2016; Rouet et al., 2016), we found that sources providing discrepant information were more often cited explicitly in an essay. Although participants did not spend more time reading discrepant texts (e.g., Hakala & O'Brien, 1995; Kammerer & Gerjets, 2014), they more often switched texts when the contents were discrepant, which we interpret as comparison processes across texts. Readers facing discrepant documents might act more strategically by comparing information across documents (see, e.g., List & Alexander, 2020). This corroborates and extends prior findings that discrepancy strongly promotes readers’ attention to sources and the integration of source and content information into a documents model (Braasch et al., 2012; Rouet et al., 2016).

Additionally, communicating information about discrepant topics needs more work—as evidenced both in longer essays and more time spent on writing the essay. To our knowledge, this extends prior findings on the DIS-C effect. The finding is all the more noteworthy because for consistent topics, four different reasons (across the two documents) covering four different aspects supporting the claim were provided, whereas for discrepant topics, the four reasons covered only two different aspects, namely two from a pro and two from a con perspective. Consequently, readers actually had more unique pieces of information to report when reading consistent versus discrepant topics. However, the participants did not seem to experience a need to write as much about consistent topics as about discrepant topics. Although an analysis of the essays with regard to the number of reasons reported would be interesting, such an analysis is also confronted with a problem that is difficult, if not impossible, to solve: How exactly to count the number of reasons participants provided, in a way that is reliable across raters. Especially in the case of discrepant topics, there are many ways of reporting reasons that are interrelated but different (i.e., complementary, contradictory), as is the case in the present study. This makes an objective counting of reasons almost impossible. However, even without this analysis, the present results suggest that reading from discrepant sources increases participants’ standard for completeness in reporting about what they read. That is, discrepant information may trigger them to report all sides of the discrepancy, whereas consistent information is not reported completely but only to an extent that readers feel that they sufficiently supported the point.

Finally, we observed an interaction of context and discrepancy on the use of adversative connectors in participants’ essays. Participants used more connectors when writing in a university context, especially when the sources provided discrepant information. This finding indicates that the participants were more likely to have represented the content as a documents model. In a situation with consistent texts, building an overall situation model is usually sufficient, and no documents model is necessary (Britt et al., 1999). In a situation with discrepant texts, however, a documents model becomes necessary, and readers might even write why they believe in one or another claim (see Rouet et al., 2016). In order to report a documents model, adversative connectors and more words overall are necessary. The results overall do not support our hypothesis that context would increase or attenuate the D-ISC effect. Rather the results suggest that both discrepancy and a university context that emphasized evaluation additively increased readers’ attention to source information and its representation.

### Influence of beliefs about science

There was a main effect of utility of science on the number of text switches: Participants with more positive beliefs more often compared information across texts. We also found interaction effects: Participants with more trust in science in the university context more often explicitly mentioned in their essay that a topic was discrepant. Also in a university context, participants with a higher perceived utility of science spent more time on the texts.

Thus, interaction effects of beliefs about science and context were in the direction that more positive beliefs boosted behavior and use of information in the university context, and not in the personal context. Positive beliefs about science may motivate students to apply academic norms when the context demands it, or else, positive beliefs about science may raise the importance of already salient academic norms in the university context. Yet, since there are only a few effects, these considerations have to be confirmed in further studies.

### The challenges of experimentally researching the context

Experimentally researching context effects poses several challenges. Ideally, participants would work in different contexts in reality. However, whereas it is quite easy to place students into a university context, it is practically difficult to collect detailed data about information processing in authentic personal contexts. We did get evidence that our manipulation was effective (for instance, through the content analysis of participants’ essays and by means of manipulation control questions), and the procedure is in line with previous research (e.g., reading from a burglar's vs. a homebuyer's perspective: Schraw et al., 1993). However, the study says little about the magnitude of the context effect as it occurs naturally, since the participants were still in the same real context: sitting at home in front of their computer, participating in a scientific study. This might have affected the results.

Second, contexts differ with regard to several aspects (see also Table 1). On the one hand this means that there is a wide variety of possible scenarios within a university context and a personal context. In addition to the two scenarios implemented in the present study, there might also be university scenarios with no audience or where academic norms are not salient and not important, whereas it is also possible to come up with personal context scenarios in which there is an audience and/or in which academic norms are salient and important. We do not assume that these two represent the defining elements of university or personal context, respectively. On the other hand, contexts are not easily manipulable such that only one context aspect is varied, since often aspects covary with other aspects. For example, in many contexts the requester is also the audience or at least part of the audience. Finding meaningful scenarios with a requester but without audience (which could then be compared to a scenario with requester and audience), is difficult. Moreover, often stakes covary with a requester and/or an audience. Thus, there is still a lot of theoretical and empirical work to do to better describe the influence of context on reading.

Related to that is the problem that it is difficult to identify causal mechanisms of context effects when several aspects of context vary at the same time. Moreover, it could also be that not one single context aspect, but a combination of different aspects (e.g., the presence of high stakes and an audience) causes an effect, but neither single variation of these aspects. Probably several methodological approaches are necessary to advance the field: While experimental research can help confirm causal explanations, ethnographic research or content analysis of real-world contributions to the social media, for example, may help identify aspects of the context or combinations of aspects of the context that are relevant and trigger different processing of multiple documents.

Likewise, future research may try to identify context differences in the actual processing of multiple documents. Such research could disentangle, for example, whether the context effects observed in the present study are caused by differences in the (construction of) the mental representation of the documents, or by differences in the presentation of this representation. With regard to discrepancies, for example, such research could also follow up whether discrepancies in the personal context are a) less likely to be noted, b) less likely to be mentally represented, or c) only less likely to be explicitly addressed in the essay (see Stadtler & Bromme, 2014). In the present study, we used only a few measures that directly addressed the process (i.e., time on texts, time on task/essay page, text switches). So, we started this kind of research, but more research is needed.

### Limitations

Apart from the limitations that arise from the challenges of context manipulation, the present study entails a number of other limitations. First, the present sample was not a representative but an ad-hoc sample. Therefore, there might be a (self-)selection of students who value science and scientific norms, which might bias results with regard to the personal context such that this sample also applies scientific norms in their personal lives. Given the high mean rating of perceived utility of science (about 4 out of 5), this might be true. However, the latter finding could also be due to our sample being a university student sample.

Second, the present experiment used a rather long procedure. As can also be deduced from the position effects we found, there might be fatigue or motivation loss effects. Yet, this should have resulted in a less careful way of working on the study, thus disguising existing context effects. Therefore, the effects we report here can be assumed to be a conservative estimation of really existing effects. On the pro side, our approach allows us to research context and discrepancy effects in a way that is less affected by topic effects, for example due to topic interest or characteristics of the specific set of texts that were used. Thus, the effects reported in the present study represent a general pattern that is not necessarily replicable in every single set of documents. Future research on multiple document comprehension should consider using several topics.

Third, future work could benefit from including also direct measures of the assumed mechanism, for example measures of activated norms, or think-aloud or eye tracking procedures to support the current interpretation of results. Also, more fine-grained log-data may be of use, for example with regard to source information. An additional limitation of our study is that the task request and the editor for writing the essay were on the same page. Therefore, we cannot disentangle time data of re-reading the task and writing the essay.

Last but not least, the procedure did not include a comprehension measure, but only behavioral and productive measures as well as a memory post-test. Further studies could also measure comprehension immediately after reading the texts. Moreover, we did not measure reading and writing skills or prior knowledge. Whereas prior knowledge effects should be reduced by our approach of using several topics, reading and writing skills may account for some variance, especially with regard to the measures that are directly derived from the essay (e.g., length, number of adversative connectors). However, given the considerable sample size and the within-participant design for the factor discrepancy, we can assume that these skills follow similar distributions across condition, such that neither context nor discrepancy effects can be explained by individual differences.

### Consequences for theory and practice

The results of the present study support the general provision of the RESOLV model (Britt et al., 2018) that the physical and social context influences reading processes. Yet, the RESOLV model only enables coarse predictions regarding which aspects of the context can make a difference and how exactly they influence reading processes and outcomes. The analysis of prior manipulations of context as well as our own manipulation show that there are several aspects intertwined inseparably across naturally occurring contexts. Further studies on the RESOLV model might want to focus on which aspects exactly contribute to a difference in the reading process. The present results suggest that the presence or absence of an audience plays a role for the presentation of information. It might also be that it is not a single aspect of the context which changes reading processes, but an additive or interactive combination of several aspects. Thus, with regard to assessing theoretical considerations about the role the context for reading, the present study took a first step, but many others are to follow.

With regard to educational practice, the present results suggest that writing in an academic context increases students’ standards for comprehending and reporting information. In addition, writing from discrepant sources increased students’ attention to sources. These findings support the use of multiple discrepant documents as a means to stimulate critical thinking and the principled integration of sources and contents in students’ essays. Thus, multiple-document assignments can support university students’ learning by promoting heuristics like corroboration or sourcing, or more generally: by promoting standards for a documents model. This is especially true if there are discrepancies across documents. While in the present research, this discrepancy was on the level of provided claims and reasons, differences at other levels, such as discrepant wording across documents, can support university students’ learning (Schoor et al., 2019).

## Conclusion

The present study provides—at least to our knowledge—the first experimental results on how different contexts influence university students’ dealing with multiple documents. A university context with salient academic norms in contrast to a personal context without this salience influences both standards of a documents model and standards for presentation. These context differences are not mitigated by positive beliefs about science, but rather enhanced. Thus, how university students approach multiple documents in their personal lives might not be affected by their university education. Further research should also consider how to foster a science-friendly approach outside the university context.

## Supplementary Information

Below is the link to the electronic supplementary material.

Supplementary file1 (PDF 183 KB)

## Footnotes

1 Due to space limitation, information about the memory measures as well as results are reported in the supplemental material.

2 Both time measures were not corrected for number of words provided in the texts. The pattern of results did not change when time data were divided by number of words.

3 The pattern of results did not change when these time data were not excluded.

4 McDonald’s ω is a measure of internal consistency alternative to Cronbach’s α, accounting for many problems of the latter (McNeish, 2017). It was calculated with the R package *psych* (Revelle, 2020).

5 This huge odds ratio is due to the fact that in the university condition, almost every essay was written as continuous text, thus this signals a ceiling effect.
