# Supplementary material for: Effects of context and discrepancy when reading multiple documents
Source: Read Writ. 2022 Jun 28;36(5):1111–43. doi: 10.1007/s11145-022-10321-2 (PMC9244203; doi:10.1007/s11145-022-10321-2)
Supplement: Supplementary file 1 — Supplementary file1 (PDF 183 KB) [file 11145_2022_10321_MOESM1_ESM.pdf]

# Supplemental Material: Memory

Schoor, C., Rouet, J.-F., Britt, M. A. (2022). Effects of context and discrepancy when reading multiple documents. *Reading and Writing*. https://doi.org/10.1007/s11145-022-10321-2

As reported in the main article, we additionally measured memory for texts, sources, and discrepancies in a post-test. In the present supplemental material, we describe the measures and the results regarding memory.

## Methods

### Post-Test: Memory for Texts, Sources, and Discrepancies

In the post-test, memory for the texts, for the sources and their claims, and for discrepancies was assessed for each of the eight target topics (based on, e.g., Maier & Richter, 2013; Stadtler et al., 2013). For each topic, six sentences were presented, two of which were distractors and four were from the texts (from each text one reason supporting the claim and one other sentence). For each of the six sentences, participants had to make decisions about a) whether or not this or a paraphrased sentence was included in the texts (yes/no), b) which was the source of this sentence (recognition: selection out of a list of the four sources that were generated per topic, two of which the participants had seen), c) whether their texts contained a statement that was discrepant with this sentence.

**Memory for Texts.** Two indices per participant were created (e.g., Maier & Richter, 2013): one representing the memory for consistent texts, and one representing the memory for discrepant texts. Both indices were calculated as the percentage of correctly identified target sentences of the respective topics (all consistent topics, all discrepant topics) minus the percentage of false-positives of the respective topics (i.e., distractor sentences incorrectly identified as being part of the texts).

**Memory for Sources and Their Claims.** For every sentence borrowed from the texts, we coded whether the participant correctly identified its source (i.e., 1 for correctly identified, 0 for not correctly identified). Note that some of the sentences in the discrepant topics were in fact not discrepant. For the analysis, consistency at the sentence level was used.

**Memory for Discrepancies.** For those topics that the participant had received in discrepant condition, we calculated the percentage of correctly identified discrepant sentences (i.e., participants correctly indicate that the sentence was part of their texts and that it was discrepant from other information provided). From this, the percentage of false-positives (i.e., consistent sentences for which participants indicated that they were part of their texts and that they were discrepant) was subtracted. Thus, an overall index for the representation of discrepant information in discrepant topics was created (see Stadtler et al., 2013).

### Data analysis

Memory data were analyzed in the same way as the other dependent measures (see main article). Since for memory for texts two scores per participant (one for consistent topics, one for inconsistent topics) were built across topic, neither topic nor position of the topic could be included in the linear mixed

model. Thus, only intercept and a random effect for the participant were included in addition to context, discrepancy and their interaction as fixed effects. With regard to memory for discrepancies, one score across all topics was built per participant. Therefore, a potential context effect in memory for discrepancies was analyzed by means of a t test in R.

## Results

Descriptive data and an overview of results for memory are displayed in Table S1. Results are illustrated in Figure S1.

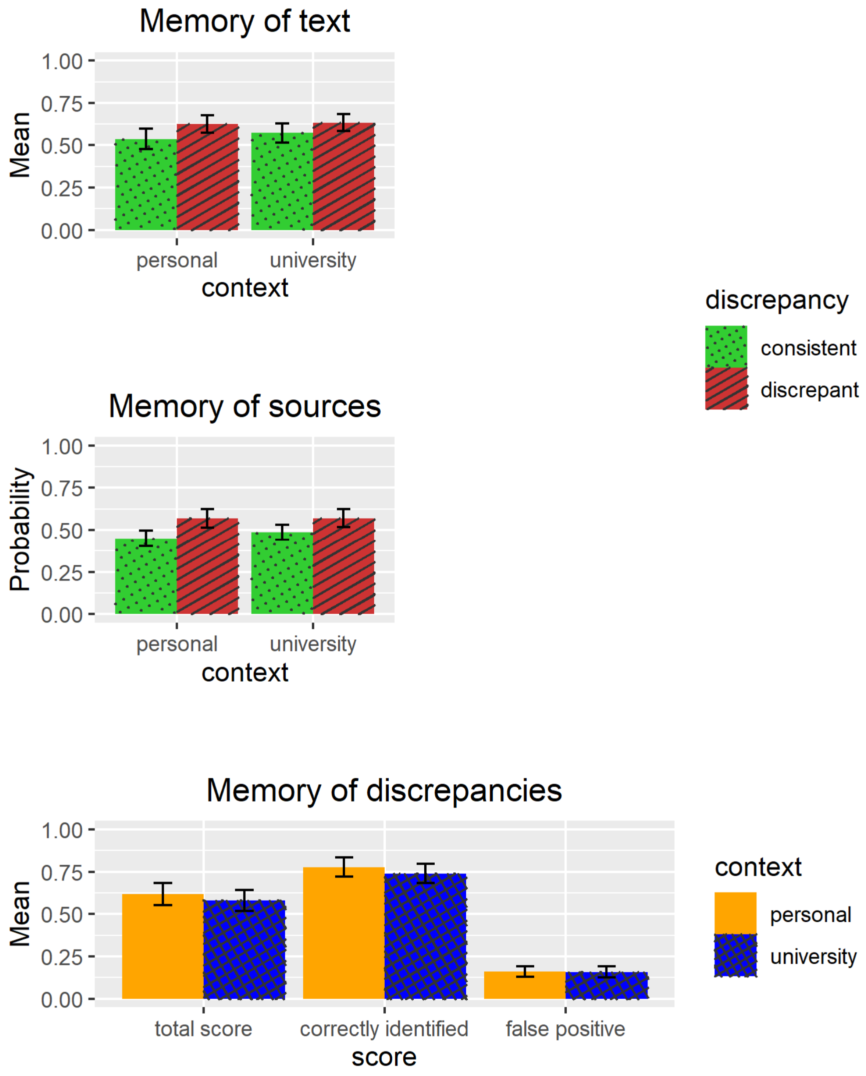


*Figure S1*. Results Regarding Memory. Error bars represent confidence intervals.

*Table S1.* Descriptive Statistics for Memory.

| | Scale Range | M (SD) | Personal consistent | Personal inconsistent | University consistent | University inconsistent | Results[a] |
|---|---|---|---|---|---|---|---|
| **Documents model:** | | | | | | | |
| Memory for texts | -1 - 1 | 0.59 (0.25) | 0.54 (0.27) | 0.63 (0.24) | 0.57 (0.26) | 0.63 (0.23) | D |
| Memory for sources | 0-1 | 0.52 (0.50) | 0.47 (0.50) | 0.63 (0.48) | 0.49 (0.50) | 0.63 (0.48) | D, Uti, P |
| Memory for discrepancies | -1 - 1 | 0.60 (0.29) | NA | 0.62 (0.30) | NA | 0.58 (0.28) | Uti |

*Note*: For memory for sources and for memory for discrepancies, the discrepancy of the respective sentences was used (not the discrepancy of the topics).
[a] Significant main and interaction effects (indicated by *) of context (C), discrepancy (D), trust in science (trust), and perceived utility of science (uti). Interactions of beliefs about science with discrepancy have not been analyzed. If there was a significant position (P) effect in the baseline model, position was controlled for in the analyses. Since memory for texts and memory for discrepancies was measured with one single score each, no position effect could be analyzed.

## Memory as an indicator of the standard for a documents model

With regard to memory for texts, we found a significant main effect of discrepancy such that information from discrepant topics was better remembered than that from consistent topics ($\beta = .17$, $p = .003$). No effect of context, interaction of context and discrepancy, effects of beliefs nor interaction of beliefs and context was found. With regard to the memory for sources, we found a main effect of discrepancy such that sources of discrepant sentences were remembered more often as compared to sources of consistent sentences (OR = 2.23, $p < .001$). Neither an effect of context nor an interaction effect of context and discrepancy was found. Trust in science had no effect on memory for sources, but there was a main effect of perceived utility of science on memory for sources (OR = 1.21, $p = .014$) that failed to reach significance when the interaction with context was included in the model. With regard to memory for discrepancies, we found no effect of context ($t$ (158) = 0.79, $p = .430$). Trust in science had again no effect but there was again a main effect of perceived utility of science on memory for discrepancies ($\beta = .06$, $p = .006$) that failed to reach significance when the interaction with context was added.

## References

Braasch, J. L. G., Rouet, J.-F., Vibert, N., & Britt, M. A. (2012). Readers' use of source information in text comprehension. *Memory & Cognition, 40*(3), 450-465. https://doi.org/10.3758/s13421-011-0160-6

Kammerer, Y., & Gerjets, P. (2014). Quellenbewertungen und Quellenverweise bei Lesen und Zusammenfassen wissensbezogener Informationen aus multiplen Webseiten [Source evaluations and source references when reading and summarizing science-related information from multiple web pages]. *Unterrichtswissenschaft, 42*(1), 7-23.

Kammerer, Y., Kalbfell, E., & Gerjets, P. (2016). Is this information source commercially biased? How contradictions between web pages stimulate the consideration of source information. *Discourse Processes, 53*(5-6), 430-456. https://doi.org/10.1080/0163853x.2016.1169968

Maier, J., & Richter, T. (2013). Text belief consistency effects in the comprehension of multiple texts with conflicting information. *Cognition and Instruction, 31*(2), 151-175. https://doi.org/10.1080/07370008.2013.769997

Rouet, J.-F., Le Bigot, L., Pereyra, G. d., & Britt, M. A. (2016). Whose story is this? Discrepancy triggers readers' attention to source information in short narratives. *Reading and Writing, 29*(8), 1549-1570. https://doi.org/10.1007/s11145-016-9625-0

Stadtler, M., Scharrer, L., Brummernhenrich, B., & Bromme, R. (2013). Dealing with uncertainty: Readers' memory for and use of conflicting information from science texts as function of presentation format and source expertise. *Cognition and Instruction, 31*(2), 130-150. https://doi.org/10.1080/07370008.2013.769996
